# Management of Pediatric Supination-inversion Ankle Injuries Involving Distal Tibia and Intraepiphyseal Distal Fibula Fractures

**DOI:** 10.5435/JAAOSGlobal-D-23-00284

**Published:** 2024-04-29

**Authors:** Jacob Shermetaro, David Sosnoski, Wendy Ramalingam, Junichi Tamai

**Affiliations:** From the Department of Orthopedic Surgery, Cincinnati Children's Hospital Medical Center, Cincinnati, OH (Dr. Shermetaro, Dr. Sosnoski, Dr. Ramalingam, and Dr. Tamai), and the Department of Orthopedic Surgery, Corewell Health, Farmington Hills, MI (Dr. Shermetaro and Dr. Sosnoski).

## Abstract

Pediatric ankle fractures are prevalent injuries that make up a notable portion of all periphyseal injuries. The Salter-Harris classification is the most popular classification about physeal and periepiphyseal injuries. Ogden expanded on this and described type 7 fractures which are completely intraepiphyseal and include propagation of the fracture from the articular surface through the epiphyseal cartilage and do not involve the physis. These injuries are common about the distal fibula in pediatric patients with supination-inversion type injuries. There are no specific guidelines or recommendations on treatment of these injuries in the literature. We present three cases of this injury pattern and describe our chosen management that leads each patient to full, painless ankle range of motion and return to all prior activities and sports without complication. Supination-inversion type pediatric ankle fractures are common injuries that all orthopaedic surgeons will encounter at some point throughout their practice or training. Recognizing fracture variants and understanding treatment options of pediatric ankle fractures are important for the orthopaedic community as a whole.

Pediatric ankle fractures are prevalent injuries that make up approximately 17% of all periphyseal pediatric injuries.^1^ The Salter-Harris classification initially proposed in 1963 is the most common system used to describe physeal and epiphyseal injuries.^2^ As an addition, Ogden proposed a classification scheme in 1981 including four supplemental growth mechanism injury patterns not previously reported.^3^ This includes the Type VII injury pattern, which describes an all-intraepiphyseal fracture, propagating from the articular surface through the epiphyseal cartilage, however not involving the physis.^3,4^ These injuries are reported to be more common in the malleoli, distal humerus, and distal femur.^3^

Regarding the ankle specifically, the scheme proposed by Dias and Tachdjian classifies pediatric ankle fractures about the position along with the mechanism and is commonly used to guide treatment in the field today.^5^ It has been reported that of these subclassifications, the most common mechanism is the supination-inversion type injury.^5,6^ Although not specifically reported in the original classification scheme, the Type VII fracture of the distal fibula is commonly the result of a supination-inversion injury about the ankle.^7^ This injury has been described previously in the literature; however, it is largely about an isolated distal fibula fracture. Several studies have also focused on a resultant “os subfibulare”, or an anterior talofibular ligament avulsion sequela, as well.^7,8,9,10^

Historically, isolated type VII injuries to the distal fibula have been successfully treated conservatively with various weight-bearing restrictions and immobilization; however, treatment of these injuries in conjunction with an ipsilateral distal tibia fracture has not been reported.^7,8,9,10^ We present three unique cases involving this injury pattern with our chosen management and successful outcomes of each.

## Statement of Informed Consent

Each patient and their families were informed that the data concerning each of their cases would be submitted for publication, and informed consent regarding release of Personal Health Information was signed by the patient's parents and obtained.

## Case Report

**Figure 1:**
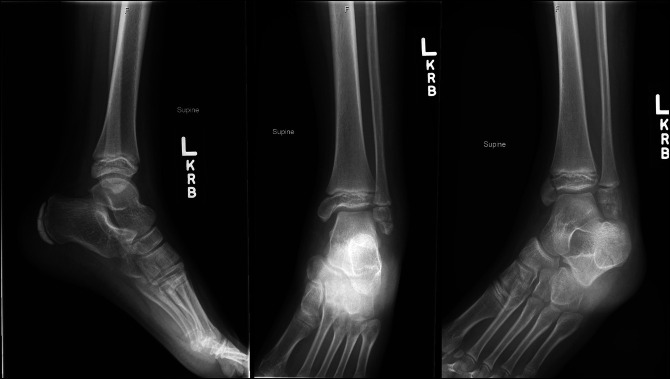
AP, mortise, and lateral view ankle radiographs of Case 1 after their injury demonstrating a displaced Salter-Harris type III medial distal tibia fracture and Ogden Type VII distal fibula fracture.

Case 1 involves a 9-year-old girl who presented 3 days after a left ankle twisting injury while jumping on a trampoline. She reported of pain and swelling with inability to bear weight. Clinical examination revealed swelling about the ankle with tenderness over the bony malleoli. Passive range of motion was limited secondary to pain and swelling. Left ankle radiographs demonstrated a Salter-Harris type-III displaced fracture of the distal tibia and an Ogden type VII displaced transverse fracture through the distal fibula epiphysis (Figure 1). The patient and family elected for surgical management which took place 5 days after the patient's injury.

**Figure 2:**
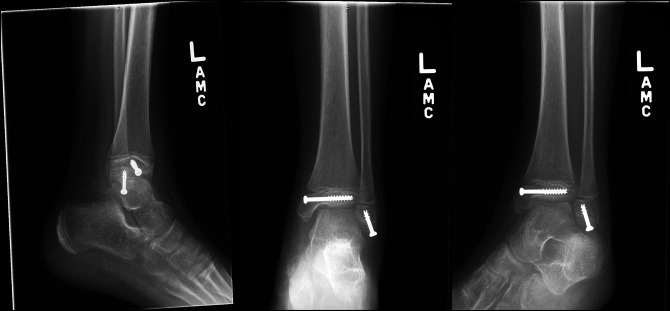
Postoperative AP, mortise, and lateral view ankle radiographs after surgical fixation.

Closed reduction with a percutaneous screw fixation technique was used as shown in Figure 2. A proper, standard technique was used to achieve anatomic articular fixation of the tibia. The distal fibula epiphysis fracture was closed reduced, and a partially threaded screw was placed to compress at the fracture site. The screw was left short of the physis to avoid iatrogenic physeal injury.

**Figure 3:**
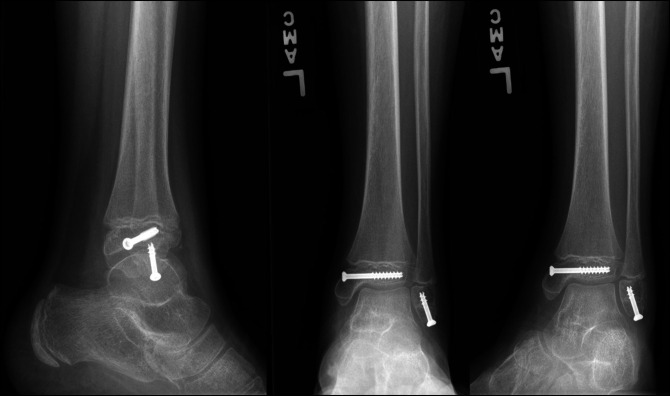
AP, mortise, and lateral view ankle radiographs 2 months after surgical fixation demonstrating complete healing of the fractures.

Postoperatively, she remained non–weight bearing in a below knee fiberglass cast for 4 weeks. At 4-week follow-up, ankle radiographs were taken showing initial bony healing at the fracture sites without physeal bar. She was subsequently transitioned to weight bearing as tolerated in a fracture boot. At 12-week follow-up, her fracture was radiographically healed (Figure 3). She demonstrated full, painless ankle plantar flexion and dorsiflexion 10° past neutral at 6-month follow-up and released to full activities including competitive soccer.

**Figure 4:**
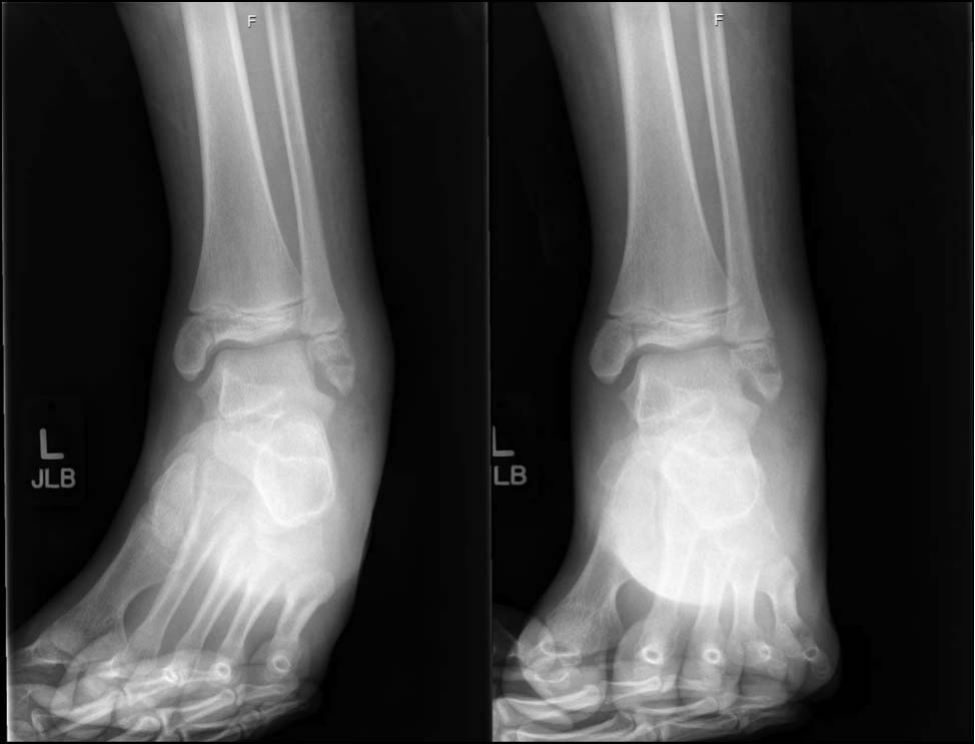
AP and mortise view ankle radiographs of Case 2 after their injury, demonstrating a displaced Salter-Harris type III medial distal tibia fracture and Ogden Type VII distal fibula fracture.

Case 2 involves a 12-year-old boy who sustained a left ankle injury while jumping on a trampoline as well. On clinical examination 3 days after injury, he had swelling about the left ankle with tenderness and limited range of motion secondary to pain. Radiographs demonstrated a displaced Salter-Harris type III medial distal tibia fracture and a displaced Ogden Type VII distal fibula fracture (Figure 4). After discussion, it was elected to proceed with surgery 6 days after the injury.

**Figure 5:**
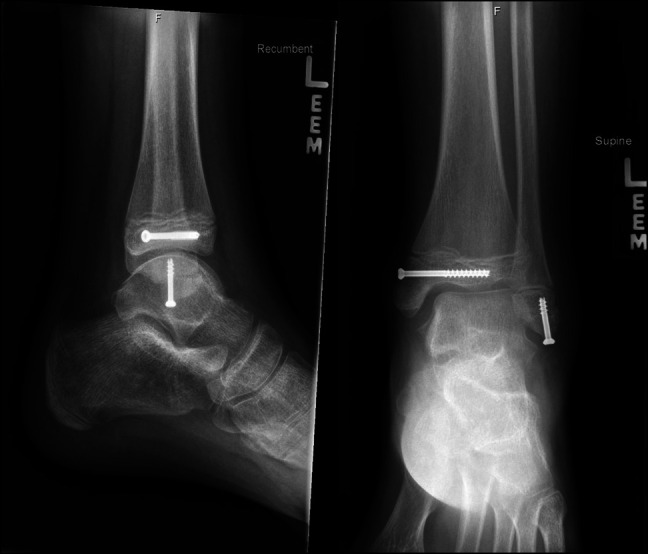
Postoperative AP and lateral view ankle radiographs after surgical fixation.

The patient underwent closed reduction with percutaneous screw fixation of left distal tibia fracture using a cannulated, partially threaded screw parallel to the physis. After attempted unsuccessful closed reduction maneuvers, the distal fibula fracture required open reduction and internal fixation with a partially threaded screw, as shown in Figure 5. It was found that the distal fibula periosteum had been interposed, therefore blocking anatomic reduction of the fracture.

**Figure 6:**
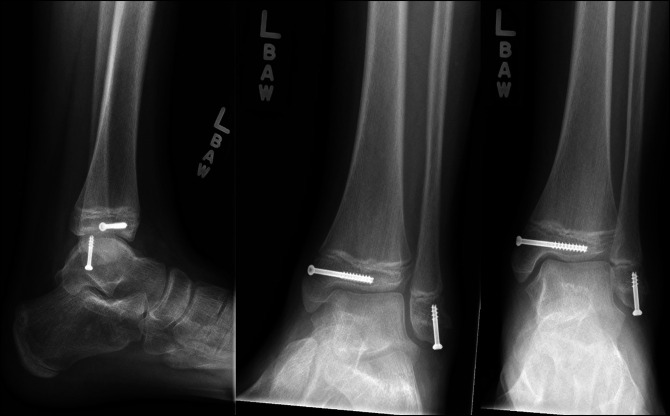
AP, mortise, and lateral view ankle radiographs 2 months after surgical fixation demonstrating complete healing of fractures.

Postoperatively, the patient remained non–weight bearing in a below-knee fiberglass cast for 4 weeks. On his 4-week postoperative visit, ankle radiographs were taken showing maintained alignment of fractures and initial bony healing seen at the fracture sites without physeal bar. He was then transitioned to weight bearing as tolerated in a fracture boot. At 12-week follow-up, radiographs demonstrated full bony healing (Figure 6). He demonstrated full, painless ankle range of motion at 6-month follow-up and was released to full activities at that time.

**Figure 7:**
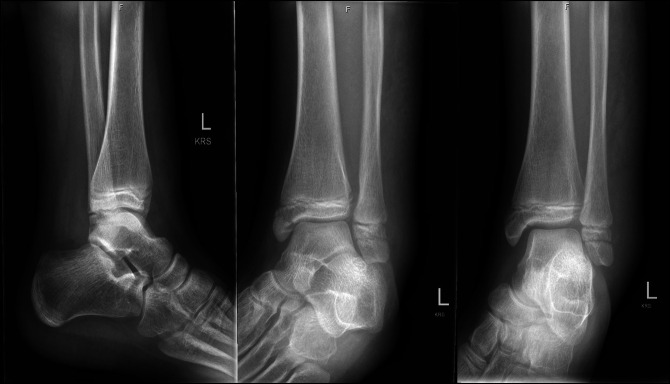
AP, mortise, and lateral view ankle radiographs of Case 3 after their injury, demonstrating a nondisplaced Salter-Harris type III medial distal tibia fracture and Ogden Type VII distal fibula fracture.

**Figure 8:**
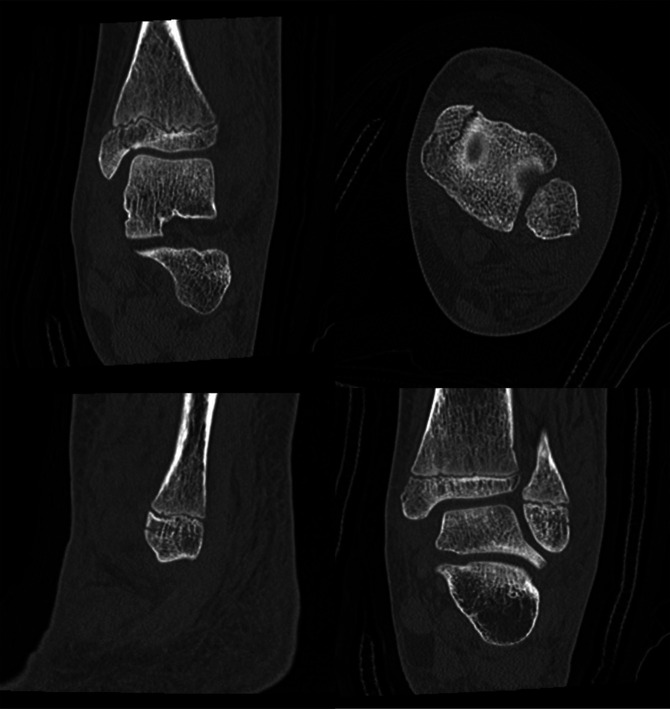
Multiple ankle CT images further demonstrating the nondisplaced fractures of Case 3.

**Figure 9:**
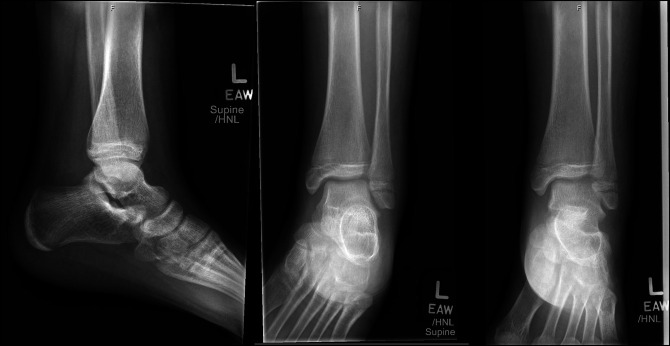
AP, mortise, and lateral view ankle radiographs 2 weeks after the injury demonstrating maintained alignment with no fracture displacement.

**Figure 10:**
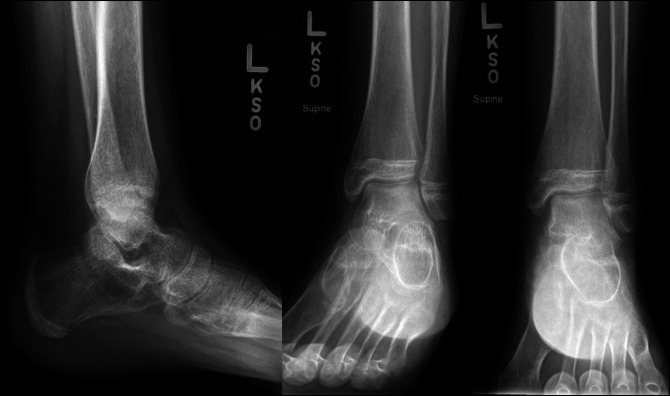
AP, mortise, and lateral view ankle radiographs 12 weeks after the injury demonstrating complete healing of fractures.

Finally, Case 3 involved a 13-year-old girl who sustained a left ankle injury after a slip and fall during gymnastics 6 days before evaluation. On examination, she had moderate swelling and ecchymosis about the ankle with tenderness over the bony malleoli. Left ankle x-rays were taken and demonstrated a nondisplaced Salter-Harris type III medial distal tibia fracture and a nondisplaced Ogden Type VII distal fibula fracture (Figure 7). A CT scan was then ordered to further evaluate potential fracture displacement (Figure 8). The CT scan confirmed adequate alignment of the fractures; therefore, nonsurgical management was pursued with non–weight bearing in a below knee fiberglass cast for 4 weeks. Radiographs taken 2 weeks postinjury demonstrated maintained fracture alignment without displacement (Figure 9). Radiographs taken 12 weeks postinjury demonstrated full bony healing of fractures without displacement (Figure 10). The patient was allowed to resume her competitive gymnastic activities and did well with this. At 6-month follow-up, she demonstrated full ankle range of motion near symmetric to the contralateral ankle without pain.

## Discussion

Ankle fractures are common injuries to the lower extremities in the pediatric population, accounting for 5% of all pediatric fractures.^11^ However, the intraepiphyseal distal fibula fracture resulting from the common supination-inversion mechanism as previously described by Ogden is much less common and requires special attention to obtain fracture healing.^3,4,9^ Previous studies with adult populations have demonstrated that distal fibula fracture malunion can result in alterations in ankle joint contact pressures, joint mechanics, and eventually causing chronic pain and premature osteoarthritis; making it essential for anatomic reduction of each.^12,13^ It has been reported that ankle arthritis later in life is secondary to posttraumatic reasons in 70% of adult patients.^12-14^

The pediatric intraepiphyseal Type VII distal fibula fracture must also be distinguished from other common ailment in pediatric patients, such as an ankle sprain. Previous reports have described the “os subfibulare,” which is a sequelae from a distal fibula fracture resulting from a sleeve avulsion of the anterior talofibular ligament.^8,10^ Failure to distinguish a type VII distal fibula fracture from an ankle sprain with an associated os subfibulare can lead to additional injury because this can result in altered ankle mechanics and additional ankle instability.^15^ One study involved two pediatric cases both with distal fibula avulsion fractures from an ankle twisting injury that went on to develop a nonunion with continued pain and ankle instability in which both patients required surgical fixation to recreate the lateral ligamentous complex.^16^

Sugi et al also sought to distinguish the os subfibulare from a type VII distal fibula fracture with radiographic parameters. They described radiographic characteristics such as long irregular fracture lines within the middle third of the distal fibular epiphysis and wider fragments are seen in type 7 fractures versus smooth-edged ossicles of shorter length about the inferior pole of the epiphysis.^10^ Although critical to the diagnosis, they did not address the management of these rare fractures, especially occurring with an associated distal tibia fracture.

In the pediatric patient, conservative treatment with casting and non–weight bearing is the preferred treatment for patients with nondisplaced, closed ankle fractures.^1,17^ It has been widely recognized that distal tibia fractures with greater than 2 mm of articular displacement require surgical fixation to avoid posttraumatic degeneration.^1,11^ Anatomic reduction of a distal tibial physeal fracture is essential in preventing physeal arrest or bar formation, and long-term follow-up should be considered.^18^ Although the Type VII distal fibula fracture is completely extraphyseal and therefore should not raise concern of possible physeal arrest and growth disturbance, anatomic fixation is quintessential.^7,14^ It is when it comes to an associated distal fibula fracture with concomitant distal tibia fracture, they are often treated surgically if displaced to establish length or to provide additional fixation.^17^ Classically, isolated type VII injuries to the distal fibula have been treated conservatively with various weight-bearing restrictions and immobilization.^7,8,9,10^ Gamble et al^9^ discussed that these isolated injuries heal well with conservative management; however, very distal fractures may be at increased risk of forming an os subfibulare and therefore subsequent ankle pain or instability with nonsurgical management.

To our knowledge, the three cases presented are the first to describe an associated Type VII intraepiphyseal distal fibula fracture with associated Salter-Harris type III distal tibia fracture. This represents a gap in the literature, and additional studies with larger patient populations are necessary to fully understand these injuries and their optimal treatment. We use the treatment algorithm starting with conservative management for nondisplaced fractures, followed by successful surgical management for displaced fractures. Recognizing these unique fracture variants is crucial in appropriately treating to return pediatric patients to their daily activities in an expedient fashion and to avoid posttraumatic arthritis in the long-standing future.
